# Post-COVID-19 Impairment of the Senses of Smell, Taste, Hearing, and Balance

**DOI:** 10.3390/v14050849

**Published:** 2022-04-20

**Authors:** Sonja Ludwig, Angela Schell, Michelle Berkemann, Frederic Jungbauer, Lena Zaubitzer, Lena Huber, Christian Warken, Valentin Held, Alexander Kusnik, Andreas Teufel, Matthias Ebert, Nicole Rotter

**Affiliations:** 1 Department of Otorhinolaryngology, Head and Neck Surgery, University Hospital Mannheim, Medical Faculty Mannheim, University of Heidelberg, Theodor-Kutzer-Ufer 1-3, 68167 Mannheim, Germany; angela.schell@umm.de (A.S.); michelleb91@web.de (M.B.); frederic.jungbauer@umm.de (F.J.); lena.zaubitzer@umm.de (L.Z.); lena.huber@umm.de (L.H.); christian.warken@umm.de (C.W.); nicole.rotter@umm.de (N.R.); 2 Department of Neurology, University Hospital Mannheim, Medical Faculty Mannheim, University of Heidelberg, Theodor-Kutzer-Ufer 1-3, 68167 Mannheim, Germany; valentin.held@umm.de; 3 Department of Medicine II, University Hospital Mannheim, Medical Faculty Mannheim, University of Heidelberg, Theodor-Kutzer-Ufer 1-3, 68167 Mannheim, Germany; alexander.kusnik@rochesterregional.org (A.K.); andreas.teufel@umm.de (A.T.); matthias.ebert@umm.de (M.E.); 4 Department of Internal Medicine, Rochester Regional Health, Unity Hospital, 1555 Long Pond Rd, Rochester, NY 14626, USA

**Keywords:** COVID-19, SARS-CoV-2, sensory system, neurological disorders

## Abstract

Background: Various symptoms have been associated with COVID-19, but little is known about the impacts of COVID-19 on the sensory system, risk factors, and the duration of symptoms. This study assesses olfactory, gustatory, hearing, and vestibular systems after COVID-19. Methods: This cross-sectional, single-center study involved 50 patients one to six months after COVID-19 and reports their patient records and the extent, onset, and duration of olfactory, gustatory, hearing, and balance disorders using questionnaires during and after COVID-19. Sensory symptoms were objectively studied using the following clinical tests after COVID-19 Sniffin’ Sticks, taste tests, tone/speech audiometry, and video head impulse test. Results: Post-COVID-19-patients were suffering from olfactory and gustatory impairment for up to six months. According to the Dizziness Handicap Inventory, balance disorders were less noticed: Overall, about 40% of the patients during COVID-19 and nearly all patients recovered within six months. After COVID-19, clinical tests revealed that 75% were suffering from hyposomnia/anosmia, and 20% of all patients reported mild hypogeusia for up to six months. Vestibular disorders and hearing impairment rarely/did not occur. Females were significantly more affected by sensory impairments than males. Conclusions: COVID-19 particularly caused olfactory and gustatory impairment; balance disorders were present too; vestibular and auditory symptoms were negligible.

## 1. Introduction

In December 2019, a novel coronavirus—severe acute respiratory syndrome coronavirus 2 (SARS-CoV-2)—emerged, causing a cluster of acute pulmonary syndromes in Wuhan, China [1]. The subsequent COVID-19 rapidly grew into a pandemic, with 80.87 million cases and 1.77 million deaths worldwide as of December 2020 [2]. Symptoms associated with COVID-19 range from mild (fever, headache, dizziness, fatigue, dry cough) to severe disease (pneumonia, acute respiratory distress syndrome, cardiogenic complications) [3,4]. Since sensory systems are affected, reports include not only olfactory and gustatory symptoms but also neurological disorders [5,6,7]. Many patients with COVID-19 feel dizzy [8]; however, it remains unclear whether this symptom reflects the effects of SARS-CoV-2 on the vestibular system or whether the dizziness has another cause. The affecting of motor cranial nerves by other viruses has been well-described (e.g., facial nerve palsy as part of a herpes zoster infection) [9]. The mechanisms and durations of the manifold neurological symptoms of COVID-19 remain unclear, as do the pathogeneses that might underlie them. Direct toxic damage to the central nervous system, and immunological and hypoxic mechanisms, have been discussed in relation to SARS-CoV-2 [10].

This study aimed at objectifying perceived or even unnoticed sensory deficits of the olfactory, gustatory, hearing, and vestibular systems after COVID-19 using the latest clinical diagnostics and structured questionnaires to compare symptoms both during and after the diagnosis of COVID-19. We sought to understand how long sensory deficits persist and whether certain patient characteristics were linked to more severe disease courses.

## 2. Materials and Methods

### 2.1. Patients

This study included 50 post-COVID-19 patients from 1 May to 30 November 2020 at the Department of Otorhinolaryngology, Head and Neck surgery. The inclusion criteria were adulthood (>18 years), being six months post-diagnosis of COVID-19, as confirmed by polymerase chain reaction (PCR) from swab samples, and approval by the local health offices. All patients included in this study had suffered from wild type virus. Prior to clinical testing, a clinical examination, including ear microscopy and nasal endoscopy, was performed. This study was approved by the local ethics committee (#2020-549N) and conducted according to the Declaration of Helsinki; all patients gave written informed consent to participate. “During COVID-19” refers to the time period of COVID-19, which was confirmed by a PCR test as mentioned above.

### 2.2. Smell Test

Olfactory testing was assessed using an extended 32-item Sniffin’ Sticks test (Burghart), as described previously [11]. Sniffin’ Sticks are felt tip pens that carry odors to retrieve a combined threshold, discrimination, and identification (TDI) score. Olfactory thresholds were examined at 16 different concentration levels. In an alternative forced choice (AFC) method, the blinded subjects were asked to identify the odors of three pens. After two correct choices, the concentration was decreased, if not, the concentration was increased. The odor threshold is the average of the last 4 of 7 total turning points.

Odor discrimination was determined using 16 sets of three odor-containing pens. Subjects were presented three pens, two with an identical scent and one with a different scent. Subjects had to identify which pen had the different scent and indicate the contrasting odor scent again in an AFC-setting.

In the odor identification task, 16 different odors had to be identified, and four different options were presented for each of the 16 odors. The correct choices of all three subtests were combined for the TDI score, making a maximum score of 48 points. Smell was categorized accordingly: anosmia ≤ 15 points, hyposmia 15–31 points, normosmia ≥ 31 points.

### 2.3. Taste Test

To assess the gustatory function, a standardized taste test was performed. The solvents for four taste qualities (sweet, sour, salty, bitter) were freshly prepared in distilled water (saccharum: 10%; acetic acid: 5%; sodium chloride: 10%; quinine chloride: 1%; as a control for trigeminal testing: raspberry with cherry). Then, 2–3 droplets of the solutions were administered to the subject’s tongue. Subjects had to identify the taste in an alternative forced choice setting.

### 2.4. Pure Tone Audiometry (PTA) and Speech Audiometry

A pure-tone average was calculated across 250, 500, 1000, 2000, 4000, and 6000 Hz bone-conduction and across 125, 250, 500, 1000, 2000, 4000, 6000, 8000, and 10,000 Hz air-conduction for each side and averaged for both sides. Additionally, pure-tone audiograms were averaged for low, medium, and high frequencies and both sides and analyzed for bone conduction (low: 250–500 Hz, medium: 1000–4000 Hz, high: 6000 Hz) and air conduction (low: 125–500, medium: 1000–4000 Hz, high: 6000–10000 Hz). The Freiburg speech intelligibility test was used to determine speech perception thresholds based on two-digit numbers and the ability to discriminate speech at suprathreshold presentation levels based on monosyllabic nouns [12]. Both tests were carried out in a soundproof cabin with regularly calibrated devices.

### 2.5. Video Head Impulse Test (vHIT)

To assess the horizontal semicircular canal function, vHIT was performed using a video oculography system (Autronic) with a high-speed infrared camera and an accelerometer to record head and ocular movement at a sampling rate of 250 Hz [13]. The vestibular ocular reflex was measured during short, horizontal head rotations/impulses with aimed head rotations at 100 to 200/s and 5° and 15° (center to lateral position). A minimum of 15 impulses per side were tested to acquire mean gains at 40, 60, and 80 ms and catch-up saccades. Results were interpreted as normal if the median gain was >0.8 and no reproducible catch-up saccades could be identified.

### 2.6. Subjective Visual Vertical (SVV) Test and Vestibular Evoked Myogenic Potentials (VEMPs)

SVV tests are a psychophysical measure of the angle between gravitational vertical and perceptual vertical that were preformed to screen for otolith dysfunction. Healthy individuals with normal otolith function align the SVV within two degrees of true vertical (0 degrees) [14]. If SVV tests showed abnormal results, then ocular and cervical VEMP testing were performed to further identify and objectify otolith dysfunction, as previously described [15].

### 2.7. Questionnaires

All subjects were asked to complete a self-constructed questionnaire regarding sensory symptoms at the time of presentation after COVID-19 recovery and during COVID-19, retrospectively. To evaluate the effects on sensory systems, all subjects categorized their smell or taste impairment according to time of appearance; and in quantitative (visual analogue scale (VAS) ranging from 0—no impairment to 10—complete loss of smell/taste) and qualitative manners (open question for type of smell or taste impairment, such as loss of senses or confused or diminished smell and taste). The subjective impairments of the vestibular and auditory systems were assessed for the onset and duration of dizziness and hearing impairment. For the evaluation of vestibular symptoms, subjects filled out the Dizziness Handicap Inventory (DHI) questionnaire for two points in time both during and after COVID-19 [16]. The DHI consists of 25 items evaluating triggers and the impact of dizziness on private social life and work life. Nine items for each area evaluate the functional and emotional aspects of dizziness, and the remaining seven items evaluate physical aspects; answers were “yes”, “no”, and “sometimes”. The calculated point score indicates the severity of the handicap (16–34 mild, 36–52 moderate, >54 severe disability) [16].

### 2.8. Statistical Analysis

Data were summarized using descriptive statistics in total numbers (N), percentages, arithmetic means, and standard deviation (SD) using IBM SPSS (version 20.0, Armonk, NY, USA) and GraphPad Prism (version 8, San Diego, CA, USA). An unpaired t-test was used for statistical analysis of parametric data, and for nonparametric data the Mann–Whitney U test or the Wilcoxon test was performed. Confidence intervals are listed along with significance tests in Table S1. Questionnaires and clinical tests were examined for correlation using Pearson correlation coefficient (Table S2). If data were missing, the patient was excluded from the corresponding analysis. To demonstrate a moderate effect (Cohen’s = 0.4) for 2 paired samples, a sample size of 50 has sufficient power (assuming alpha = 0.05 and power = 0.8). This has been verified using the SAS procedure PROC POWER. A *p*-value below 0.05 was considered statistically significant.

## 3. Results

### 3.1. Patient Characteristics

All subjects were recruited 1–6 months following COVID-19 confirmation by a positive PCR swab test result. In total, 23 men and 27 women with an average age of 45 years participated (Table 1). Of all patients, 88% were treated on an outpatient basis, 6% received inpatient care, and 4% received intensive care. Symptoms associated with COVID-19 persisted for 19 days on average. The consumption of noxious agents, such as tobacco or alcohol, was not the norm (tobacco: 2%; alcohol: 34%). Hypertension and thyroid dysfunction were the most common comorbidities.

### 3.2. Presence and Duration of Sensory Dysfunction after COVID-19

Sensory impairment of COVID-19 patients was assessed using questionnaires. The presence, exact beginning, and duration of symptoms are summarized in Table 2. Most patients retrospectively reported that they had sensory COVID-19-associated symptoms which affected smell (78%), taste (84%), hearing (16%), and balance (48%).

The beginning of sensory symptoms after diagnosis was similar (olfactory: 4.9 days, gustatory: 3.7 days, balance: 3.2 days, hearing: 2.9 days after diagnosis). However, many patients were unable to identify when their symptoms began.

The presence of olfactory and gustatory symptoms was frequently limited to ongoing cases of COVID-19 (olfactory: 30%; gustatory: 42%), but often continued (olfactory: 42%; gustatory: 38%). While dizziness was common, particularly during COVID-19 (30%) and even afterwards (10%), hearing was only impaired during and/or after COVID-19 in a relatively small number of cases (8%). The mean duration of olfactory and gustatory symptoms was 17–20 days. Vestibular and hearing impairments lasted 11–12 days on average.

Some patients (16%) reported pre-existing gustatory and olfactory impairments before COVID-19. A minority of subjects (olfactory: 4%; gustatory: 2%) described their symptoms as exclusively present after COVID-19.

### 3.3. Reduced Perception of Smell and Taste during and after COVID-19

**Figure 1:**
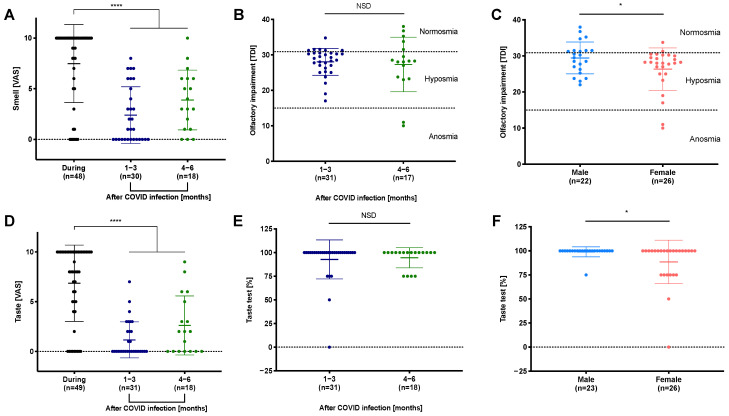
Impacts of COVID-19 on the olfactory and gustatory systems. Patients categorized their smell and taste dysfunction on a VAS (0—no impairment; 10—complete smell/taste loss) retrospectively for the time during COVID-19 and for 1–6 months after COVID-19. (**A**) Self-assessed impact on smell during and after COVID-19. The sense of smell was strongly impaired during COVID-19 and improved in the initial 6 months following COVID-19. (**B**) Olfactory impairment was measured using Sniffin’ Sticks to determine TDI scores. Most subjects suffered from hyposmia during the initial 6 months after COVID-19. (**C**) Women showed significantly stronger olfactory impairment than men. (**D**) Self-assessed taste dysfunction during infection and within the initial 6 months after COVID-19. The sense of taste partially recovered in the initial 6 months after COVID-19. (**E**) Gustatory impairment was detected via taste test and is shown as a percentage. Most patients showed fully recovered gustatory function. (**F**) Women were significantly more often affected by taste dysfunction than men; n: number of patients; *p*-values below 0.05 were considered significant (* *p* < 0.05, **** *p* < 0.0001).

Figure 1A,D shows the degrees of loss of smell and taste as rated by the patients. Olfactory and gustatory symptoms were reported by 85% and 82% of all patients, respectively, as happening during COVID-19. Olfactory disorders were frequently reported for up to 6 months (1–3 months: 53%; 4–6 months: 83% of patients); gustatory impairment lasted less frequently (1–3 months: 39%; 4–6 months: 61% of patients). While symptoms tended to persist, smell and taste were significantly reduced during COVID-19 compared to the following months (Table S1: smell/taste VAS: *p* < 0.0001). Retrospectively, 58% of all patients classified their smell disorder as complete anosmia and 45% as complete taste loss.

The objective measures of smell and taste confirmed persistent impairment in many patients after up to six months (Table 3, Figure 1B,E). TDI scores revealed that 72% of all study participants suffered from hyposmia and 4% from anosmia following COVID-19 (Table 3).

In contrast, gustatory impairment was less frequent, with 16% of patients reporting hypogeusia and 2% reporting ageusia during clinical testing (Table 3). Overall, gustatory dysfunction was less frequently observed than olfactory impairment after COVID-19. There was little variation for the different taste qualities (sweet 12%> salty 8%> bitter 4%> sour 2%). However, smell and taste did not vary between 1–3 months and 4–6 months after COVID-19 (Figure 1B,E). The subjective assessment of smell and taste (VAS) and the objective testing (i.e., Sniffin’ Sticks) showed a medium correlation for TDI scores (Table S2: TDI score: r = −0.5, *p* = 0.001).

In the objective testing for olfactory and gustatory dysfunction, female participants were significantly more affected than males (Figure 1C,F: *p* < 0.05). Interestingly, the subjective perception of smell and taste assessed using the VAS did not differ between genders (Figure S1A,B).

### 3.4. Decreased Dizziness during First Months of Follow-Up

**Figure 2:**
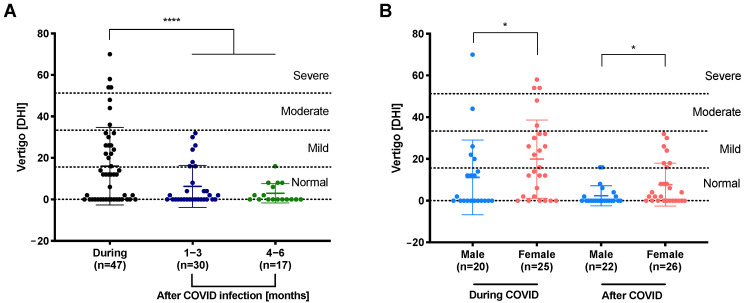
Influence of COVID-19 on the sense of balance. All patients were asked to classify their vertigo symptoms both during and after COVID-19 using the Dizziness Handicap Inventory, a 25-item multiple choice questionnaire. (**A**) During COVID-19, more than half of patients reported having at least mild symptoms of dizziness that improved during the initial six months following COVID-19. (**B**) Women experienced significantly more balance disorders than men both during and after COVID-19 (* *p* < 0.05, **** *p* < 0.0001).

The subjective perception of vertigo among the study participants was acquired using the DHI. Dizziness was common during COVID-19: 38% of patients reported mild to severe symptoms. These symptoms were less persistent, as only 23% and 6% of cases reported mild symptoms at 1–3 and 4–6 months, respectively (Figure 2A, Table S1: *p* < 0.001).

Women were more likely to experience dizziness than men (Figure 2B, *p* < 0.05), and their symptoms tended to be more severe and were more likely to persist.

### 3.5. Vestibular and Auditory Functions Rarely Affected after COVID-19

One patient showed abnormal SVV test results; cervical VEMP and ocular VEMPs were absent on the left side, indicating a left otolith dysfunction. Another patient presented with an abnormal vHIT. All other patients presented with normal otolith and semicircular canal function (Table 3, Figure S2). PTAs and Freiburg speech audiograms revealed normal to mild hearing loss. Some patients had to be excluded because of pre-existing hearing disorders. However, the frequency-specific analysis showed hearing loss particularly in higher frequencies compared to lower (bone-conduction: 36% vs. 20%, air conduction: 48% vs. 36%; Table S3). Two patients out of 50 (4%) indicated acute tinnitus during and after COVID-19.

## 4. Discussion

In our cohort, 74–80% of COVID-19 patients suffered from olfactory and/or gustatory dysfunction for at least two weeks. While the initial rates were comparable to those reported by Moein and Niklassen (96% hyposmia in COVID-19 patients), our patients tended to have prolonged symptoms. Many patients reported symptoms even after 4–6 months (hyposmia (53%), anosmia (12%), and hypogeusia (22%), as compared to 63% recovering by 4–5 weeks in the cohort of Moein et al. [17,18,19]). In other studies, most patients (70–80%) indicated complete recovery from chemosensory disorders within 1–2 months [20,21]. These comparatively high recovery rates could be due to their exclusively questionnaire-based study design without objectivization and without the need for a visit to the clinic. Hence, while their recovery rates might have been overestimated, our rates might be too low due to selection bias.

In contrary to other data, we did not observe significant differences among certain taste qualities after COVID-19, which might have been due to the flaws of self-assessment [19,22]. Additionally, taste recovery rates are much higher and faster than smell recovery rates. This could be caused by the location of the SARS-CoV-2 reservoir, which is mainly in the nose and nasal cavities, which would sustain inflammatory responses and the impairment of smell [23]. Additionally, the renewal of taste buds is usually a matter of 10 days; however, the renewal of olfactory neurons takes several weeks [24,25]. Women were more affected by smell and taste dysfunction according to their TDI scores compared to men, which has been observed by others before [26]. Gender-related differences were observed only in the objective data from the Sniffin’ Sticks or taste tests, but not in the results from the questionnaires. This result is even more interesting, since the normative data reported by Oleszkiewicz et al. showed lower TDI scores for males [27].

Overall, post-COVID symptoms have been observed to be more prevalent in women than in men in various other studies [28,29,30]. In the literature, different explanations exist for these gender-related differences: The higher expression levels of the angiotensin-converting-enzyme-2 (ACE_2_) and lower levels of pro-inflammatory cytokines (i.e., interleukin-6) in women after viral infections could explain their higher susceptibility to developing olfactory and gustatory post-COVID symptoms [30,31]. Additionally, unfavorable psychological factors, such as stress, sleep, anxiety, and depressive disorders, were observed to a greater extent in women and might also have an impact on the perception of sensory symptoms, such as dizziness [32].

The objective (TDI-scores) and subjective (VAS) data for olfactory impairment showed a strong correlation (−0.6, Table S2), whereas gustatory and balance disorders showed no correlation. Overall, olfactory impairment was underestimated and gustatory disorders were overrated in self-assessed tests compared to the objective data. The subjective overestimation of gustatory symptoms might have been caused by the misunderstanding of the difference between smell and taste within the study population, which has also been observed by others, too [22]. Direct comparisons of objective and subjective assessments of olfactory loss in COVID-19 in a systematic review also showed objective testing to be more sensitive [33]. Additionally, the DHI interrogates different origins of balance disorders; however, the vHIT specifically tests an impairment of the semicircular canal. Overall, most of the objective tests are more specific than the subjective questionnaire surveys. Thus, objective measurements should be preferred to detect even unnoticed disorders of the sensory senses; some researchers have even suggested that objective smell and taste dysfunctions are sensitive indicators of COVID-19 [34,35].

The duration of smell and taste impairment was limited due to our cross-sectional and partly retrospective study design. In 20–30% of cases, sensory symptoms were ongoing during clinical tests, and the memories of symptoms could have been distorted in retrospect. Although a longitudinal study design would be more adequate to precisely detect possible effects on the sensory organs in the course of the disease and afterwards, practical reasons and hygiene guidelines make the assessment of the tests difficult, especially during the infectious phase of COVID-19; for example, a sound-attenuated booth with regularly calibrated equipment is needed for comparable auditory and vestibular assessment.

Standardized DHI questionnaires showed dizziness was a common symptom during the acute phase of infection, but tended to improve swiftly. However, some patients reported persistent dizziness for up to six months. Women were significantly more affected by dizziness during/after COVID-19. This does not seem surprising, as peripheral vestibular disorders and unspecific dizziness are more prevalently reported by women, as women overall seem to be more susceptible to dizziness and dizziness-related reductions in quality of life [36,37,38].

Interestingly, vestibular laboratory testing and hearing assessments revealed normal vestibular and cochlear function in the study population. Hence, it seems unlikely that the dizziness experienced by patients during infection was caused by SARS-CoV-2 affecting the vestibular system itself, as a sign of compensating for loss of function would be expected in vHIT [39]. However, recently, cases of vestibular neuritis and sudden onset sensorineural hearing loss post-COVID-19 have been described [40,41,42]. Nevertheless, the pathophysiology for the deficits described in those cases vary: the sudden sensorineural hearing losses were caused by intralabyrinthine hemorrhage or vestibular neuritis [40,42,43].

There might be multiple explanations for why individuals with COVID-19 experience dizziness during infection: hyperventilation due to (subjective) dyspnea, for example, can lead to dizziness and light-headedness, which is often seen in patients with anxiety disorders [44]. Moreover, orthostatic cardiovascular factors, especially in combination with fever and potential hypovolemia, and anxiety-associated hyperventilation, might lead to dizziness. Four percent of our patients indicated acute tinnitus after COVID-19. In the meta-analysis of Jafari et al., subjective tinnitus of 4.5% was calculated for the 1.3% to 23.2% tinnitus occurrence rates of COVID-19 patients [45,46,47]. However, it is controversial if the tinnitus is induced by the virus or a psychological side effect due to the mental stress [48]. In comparing pure tone averages, we did not observe significant changes; however, the frequency-specific analysis revealed that higher frequencies are more affected than medium/lower. The reported subjective auditory loss of COVID-19 patients varied from 0.6% to 10% in previous studies [45,49]. De Souza et al. compared PTAs and frequency-specific hearing loss in COVID-19 patients with moderate to severe disease courses to primary care patients (control). They observed mild to moderate hearing loss, particularly for frequencies above 1 kHz, which is in line with our observation [50].

Nevertheless, our results suggest that SARS-CoV-2 does not seem to affect the peripheral end organs of the inner ear itself, as no altered vestibular function was observed, nor was any significant loss of hearing found in study participants.

As the nasal cavities and pharyngeal mucosa are considered as SARS-CoV-2 reservoirs, SARS-CoV-2 is hypothesized to enter through the neural–mucosal interface of the olfactory mucosa. Interestingly, in animal models the sustentacular cells within the olfactory epithelium have been targeted by the virus [23,51] and the angiotensin-converting enzyme 2 (ACE_2_) has been identified as the main entrance receptor for the uptake of SARS-CoV-2 [52,53]. The infection of the olfactory epithelium with the virus causes cell death and desquamation, which can lead to a loss of olfactory neurons and consequently hyposomnia or anosmia. Whereas the affecting of the olfactory nerve has been investigated carefully, only a few studies have hypothesized about the causes for the effects in other sensory systems: (i) The first hypothesis is that sensory cells are impaired in their functions because of inflammatory processes. Particularly, in the airway epithelium, interferon and toll-like receptors are overexpressed. Additionally, higher interferon expression is associated with overexpression of ACE_2_ receptors, which in turn makes the cells more vulnerable [54,55]. (ii) The other hypothesis is that the virus spreads along the axons to other locations of the central nervous system [56]. Other cranial nerves in the brain stem could be affected [57]. Thus, caudal cranial nerves are less often involved due to their rather distant location from the olfactory nerve. However, this is controversial because losses of sensory functions in patients are usually associated with positive disease progression without encephalitis [58]. Nevertheless, it would explain the occurrence of auditory and vestibular symptoms in some patients. Thus, it is not surprising that the neuronal effects of SARS-CoV-2 on the olfactory and gustatory systems have appeared frequently, whereas malfunctions of the vestibulocochlear nerve are rare.

Our study limitations: Firstly, our sample size was—although statistically sufficient—rather small, with 50 patients included. Additionally, only patients who had suffered from SARS-CoV-2 wild type infection were included. A bigger sample size and especially patients who had undergone a virus variant infection would have been interesting to include. Presently, there unfortunately is still no literature available that compares or examines the influences of different SARS-CoV-2 virus variants on the sensory system. This would be of special interest, as potential differences are certainly conceivable, since patients infected with omicron overall suffer from other/milder symptoms than patients infected with wild type virus or the delta variant [59]. The top five symptoms that are linked to an omicron infection, for example, do not include sense of smell or taste, which were most common with the alpha variant [60]. Therefore, future studies should also include objective measurements of the sensory system, in order to detect possible differences in the impacts of SARS-CoV-2 virus variants on the sensory system.

## 5. Conclusions

Our data confirm a high incidence of impaired olfactory and gustatory function in patients during and after COVID-19. Moreover, the results suggest that the cochlear and vestibular impairment by SARS-CoV-2 is negligible, unlike the gustatory and especially the olfactory systems. Additionally, women were significantly more affected by dizziness, hypogeusia, and hyposmia compared to men. Objective measurements should be preferred to further analyze the effects of COVID-19 on the sensory system and to detect potentially unnoticed distorted functions.

## Figures and Tables

**Table 1:** Characteristics of COVID 19 patients.

Characteristics	N (%)
**Age**	
20–40 years	22 (44)
41–76 years	28 (56)
Mean ± SD	45 ± 15.44
**Gender**	
Male	23 (46)
Female	27 (54)
**Location**	
Outpatient	44 (88)
Inpatient	3 (6)
Intensive care	2 (4)
No data	1 (2)
**Duration of disease**	
1–7 days	4 (8)
8–14 days	21 (42)
15–30 days	17 (34)
30–60 days	6 (12)
No data	2 (4)
Mean ± SD	18.54 ± 11.01
**Follow up after COVID-19**	
1–3 months	32 (64)
4–6 months	18 (36)
Mean (days) ± SD	69.12 ± 41.61
**Smoker**	
Yes	1 (2)
No	49 (98)
**Alcohol consumption**	
Yes	17 (34)
No	32 (64)
No data	1 (2)
**Medical history**	
Hypertension	10 (20)
Atrial Fibrillation	2 (4)
Depression	3 (6)
Asthma	4 (8)
Thyroid dysfunction	8 (16)
**Medication**	
Hypertensive medication	11
Antilipemic medication	4
Antidepressant medication	5
Asthma medication	3
Analgesics	3
Others	21

**Table 2:** Questionnaire data of sensory dysfunction during and after COVID-19.

	Olfactory N (%)	Gustatory N (%)	Hearing N (%)	Vestibular N (%)
**Presence of symptoms**				
None Before COVID-19	8 (16) 3 (6)	8 (16) 0 (0)	34 (68) 8 (16)	23 (46) 3 (6)
*COVID19-associated symptoms:*				
Only during COVID-19	15 (30)	21 (42)	3 (6)	15 (30)
During and after	21 (42)	19 (38)	1 (2)	5 (10)
Only after	2 (4)	1 (2)	0 (0)	4 (8)
No data	1 (2)	1 (2)	4 (8)	0 (0)
Total	50 (100)	50 (100)	50 (100)	50 (100)
**Initial begin of COVID-19 associated symptoms**				
With onset of COVID-19	9 (18)	10 (20)	1 (2)	8 (16)
1–7 days after onset	20 (40)	24 (48)	4 (2)	13 (26)
8–14 days after onset	5 (10)	4 (8)	0 (0)	2 (4)
15–21 days after onset	0 (0)	0 (0)	0 (0)	1 (4)
31 days after onset	1 (2)	0 (0)		
No data	15 (30)	12 (24)	45 (90)	26 (52)
Total	50 (100)	50 (100)	50 (100)	50 (100)
Mean ± SD	4.86 ± 5.68	3.74 ± 3.29	3.2 ± 3.49	2.96 ± 4.59
**Duration of disease**				
1–7 days	9 (18)	11 (22)	1 (2)	14 (28)
8–14 days	7 (14)	10 (20)	1 (2)	2 (4)
15–21 days	4 (8)	6 (12)	1 (2)	1 (5)
22–120 days	5 (12)	7 (14)	0 (0)	4 (8)
No data	25 (50)	16 (32)	47 (94)	29 (58)
Total	50 (100)	50 (100)	50 (100)	50 (100)
Mean ± SD	19.92 ± 27.37	16.53 ± 20.4	11.33 ± 9.07	11.67 ± 14.73

**Table 3:** Clinical test data of sensory dysfunction after COVID-19 infection.

	**Olfactory Test**		**Gustatory Test**
**Sniffing sticks**		**Taste test**	
Threshold	3.03 ± 2.73	Normal taste (100%)	40 (80)
Discrimination	12.24 ± 2.38	Hypogeusia (25–75%)	8 (16)
Identification	12.63 ± 2.57	Ageusia (0%)	1 (2)
		No data	1 (2)
TDI score	27.78 ± 5.37	Total	50 (100)
Normosmia (≥ 31 pts)	11 (22)	Retronasal irritation test	
Hyposmia (15–31 pts)	36 (72)	Perceived and identified	39 (78)
Anosmia (≤15 pts)	2 (4)	Perceived	8 (16)
No data	1 (2)	No perception	3 (6)
Total	50 (100)	Total	50 (100)
	**Audiometric Test**		**Vestibular Test**
**PTA—bone conduction**		**Video head impulse test**	
Normal hearing (0–20 dB)	36 (72)	**(vHIT)**	
Mild (20–40 dB)	6 (12)	Normal	48 (96)
Medium (40–60 dB)	0 (0)	Abnormal	1 (2)
Severe (>60 dB)	0 (0)	No data	1 (2)
No data/Pre-existing	8 (16)	Total	50 (100)
Total	50 (100)	Mean ± SD	1.03 ± 0.15
Mean ± SD	11.3 ± 6.7		
**PTA—air conduction**			
Normal hearing (0–20 dB)	27 (54)		
Mild (20–40 dB)	15 (30)		
Medium (40–60 dB)	0 (0)		
Severe (>60 dB)	0 (0)		
No data/Pre-existing	8 (16)		
Total	50 (100)		
Mean ± SD	18.2 ± 8.3		
**Freiburg speech audiogram**			
Numbers at 50%	−9.56 ± 13.22		
No data	8 (16)		
Monosyllables at 65 dB	93.91 ± 13.12		
No data	10 (20)		
Total	50 (100)		
**Tympanogram**			
Normal	46 (96)		
Abnormal	3 (6)		
No data	1 (2)		
Total	50 (100)		
**Stapedius reflex test**			
Normal	22 (44)		
Abnormal	14 (28)		
No data	14 (28)		
Total	50 (100)		

## Data Availability

Not available.

## Supplementary Materials

The following supporting information can be downloaded at: https://www.mdpi.com/article/10.3390/v14050849/s1. Figure S1: The subjective impairment of smell and taste distinguished by gender. Figure S2: Video head impulse test (vHIT) after COVID infection. Table S1: Subjective and objective impairment by smell, taste and vertigo. Table S2: Correlation analysis of questionnaires and clinical tests.

## References

[1] Zhu N., Zhang D., Wang W., Li X., Yang B., Song J., Zhao X., Huang B., Shi W., Lu R. (2020). A Novel Coronavirus from Patients with Pneumonia in China, 2019. N. Engl. J. Med..

[2] Dong E., Du H., Gardner L. (2020). An interactive web-based dashboard to track COVID-19 in real time. Lancet Infect. Dis..

[3] Li L.Q., Huang T., Wang Y.Q., Wang Z.P., Liang Y., Huang T.B., Zhang H.Y., Sun W., Wang Y. (2020). COVID-19 patients’ clinical characteristics, discharge rate, and fatality rate of meta-analysis. J. Med. Virol..

[4] Kim G.U., Kim M.J., Ra S.H., Lee J., Bae S., Jung J., Kim S.H. (2020). Clinical characteristics of asymptomatic and symptomatic patients with mild COVID-19. Clin. Microbiol. Infect..

[5] Iadecola C., Anrather J., Kamel H. (2020). Effects of COVID-19 on the Nervous System. Cell.

[6] Ahmed M.U., Hanif M., Ali M.J., Haider M.A., Kherani D., Memon G.M., Karim A.H., Sattar A. (2020). Neurological Manifestations of COVID-19 (SARS-CoV-2): A Review. Front. Neurol..

[7] Simon O.J., Timmermann L. (2020). COVID-19: A neurological point-of-view. Dtsch. Med. Wochenschr..

[8] Saniasiaya J., Kulasegarah J. (2021). Dizziness and COVID-19. Ear Nose Throat J..

[9] Adour K.K., Bell D.N., Hilsinger R.L. (1975). Herpes simplex virus in idiopathic facial paralysis (Bell palsy). JAMA.

[10] Wu Y., Xu X., Chen Z., Duan J., Hashimoto K., Yang L., Liu C., Yang C. (2020). Nervous system involvement after infection with COVID-19 and other coronaviruses. Brain Behav. Immun..

[11] Hummel T., Sekinger B., Wolf S.R., Pauli E., Kobal G. (1997). ‘Sniffin’ sticks’: Olfactory performance assessed by the combined testing of odor identification, odor discrimination and olfactory threshold. Chem. Senses.

[12] Hoth S. (2016). The Freiburg speech intelligibility test: A pillar of speech audiometry in German-speaking countries. HNO.

[13] MacDougall H.G., Weber K.P., McGarvie L.A., Halmagyi G.M., Curthoys I.S. (2009). The video head impulse test: Diagnostic accuracy in peripheral vestibulopathy. Neurology.

[14] Böhmer A., Mast F. (1999). Assessing otolith function by the subjective visual vertical. Ann. N. Y. Acad. Sci..

[15] Birk R., Dietz M., Sommer J.U., Stuck B.A., Hörmann K., Rotter N., Maurer J.T., Kramer B., Hülse R., Schell A. (2020). Nightly Hypoxia Does Not Seem to Lead to Otolith Dysfunction in Patients with Obstructive Sleep Apnea. Ear Nose Throat J..

[16] Jacobson G.P., Newman C.W. (1990). The development of the dizziness handicap inventory. Arch. Otolaryngol. Head Neck Surg..

[17] Moein S.T., Hashemian S.M., Tabarsi P., Doty R.L. (2020). Prevalence and reversibility of smell dysfunction measured psychophysically in a cohort of COVID-19 patients. Int. Forum Allergy Rhinol..

[18] Niklassen A.S., Draf J., Huart C., Hintschich C., Bocksberger S., Trecca E.M.C., Klimek L., Le Bon S.D., Altundag A., Hummel T. (2021). COVID-19: Recovery from Chemosensory Dysfunction. A Multicentre study on Smell and Taste. Laryngoscope.

[19] Konstantinidis I., Delides A., Tsakiropoulou E., Maragoudakis P., Sapounas S., Tsiodras S. (2020). Short-Term Follow-Up of Self-Isolated COVID-19 Patients with Smell and Taste Dysfunction in Greece: Two Phenotypes of Recovery. ORL J. Otorhinolaryngol. Relat. Spec..

[20] Printza A., Katotomichelakis M., Valsamidis K., Metallidis S., Panagopoulos P., Panopoulou M., Petrakis V., Constantinidis J. (2021). Smell and Taste Loss Recovery Time in COVID-19 Patients and Disease Severity. J. Clin. Med..

[21] Speth M.M., Singer-Cornelius T., Oberle M., Gengler I., Brockmeier S.J., Sedaghat A.R. (2020). Time scale for resolution of olfactory dysfunction in COVID-19. Rhinology.

[22] Singer-Cornelius T., Cornelius J., Oberle M., Metternich F.U., Brockmeier S.J. (2021). Objective gustatory and olfactory dysfunction in COVID-19 patients: A prospective cross-sectional study. Eur. Arch. Otorhinolaryngol..

[23] Bryche B., St Albin A., Murri S., Lacote S., Pulido C., Ar Gouilh M., Lesellier S., Servat A., Wasniewski M., Picard-Meyer E. (2020). Massive transient damage of the olfactory epithelium associated with infection of sustentacular cells by SARS-CoV-2 in golden Syrian hamsters. Brain Behav. Immun..

[24] Beidler L.M., Smallman R.L. (1965). Renewal of cells within taste buds. J. Cell Biol..

[25] Graziadei P.P., Levine R.R., Monti Graziadei G.A. (1979). Plasticity of connections of the olfactory sensory neuron: Regeneration into the forebrain following bulbectomy in the neonatal mouse. Neuroscience.

[26] Lechien J.R., Chiesa-Estomba C.M., De Siati D.R., Horoi M., Le Bon S.D., Rodriguez A., Dequanter D., Blecic S., El Afia F., Distinguin L. (2020). Olfactory and gustatory dysfunctions as a clinical presentation of mild-to-moderate forms of the coronavirus disease (COVID-19): A multicenter European study. Eur. Arch. Otorhinolaryngol..

[27] Oleszkiewicz A., Schriever V.A., Croy I., Hahner A., Hummel T. (2019). Updated Sniffin’ Sticks normative data based on an extended sample of 9139 subjects. Eur. Arch. Otorhinolaryngol..

[28] Iqbal F.M., Lam K., Sounderajah V., Clarke J.M., Ashrafian H., Darzi A. (2021). Characteristics and predictors of acute and chronic post-COVID syndrome: A systematic review and meta-analysis. EClinicalMedicine.

[29] Yong S.J. (2021). Long COVID or post-COVID-19 syndrome: Putative pathophysiology, risk factors, and treatments. Infect. Dis..

[30] Fernandez-de-Las-Penas C., Martin-Guerrero J.D., Pellicer-Valero O.J., Navarro-Pardo E., Gomez-Mayordomo V., Cuadrado M.L., Arias-Navalon J.A., Cigaran-Mendez M., Hernandez-Barrera V., Arendt-Nielsen L. (2022). Female Sex Is a Risk Factor Associated with Long-Term Post-COVID Related-Symptoms but Not with COVID-19 Symptoms: The LONG-COVID-EXP-CM Multicenter Study. J. Clin. Med..

[31] Ortona E., Buonsenso D., Carfi A., Malorni W., Long Covid Kids study g. (2021). Long COVID: An estrogen-associated autoimmune disease?. Cell Death Discov..

[32] Salari N., Hosseinian-Far A., Jalali R., Vaisi-Raygani A., Rasoulpoor S., Mohammadi M., Rasoulpoor S., Khaledi-Paveh B. (2020). Prevalence of stress, anxiety, depression among the general population during the COVID-19 pandemic: A systematic review and meta-analysis. Glob. Health.

[33] Hannum M.E., Ramirez V.A., Lipson S.J., Herriman R.D., Toskala A.K., Lin C., Joseph P.V., Reed D.R. (2020). Objective Sensory Testing Methods Reveal a Higher Prevalence of Olfactory Loss in COVID-19-Positive Patients Compared to Subjective Methods: A Systematic Review and Meta-Analysis. Chem. Senses.

[34] Pierron D., Pereda-Loth V., Mantel M., Moranges M., Bignon E., Alva O., Kabous J., Heiske M., Pacalon J., David R. (2020). Smell and taste changes are early indicators of the COVID-19 pandemic and political decision effectiveness. Nat. Commun..

[35] Bhattacharjee A.S., Joshi S.V., Naik S., Sangle S., Abraham N.M. (2020). Quantitative assessment of olfactory dysfunction accurately detects asymptomatic COVID-19 carriers. EClinicalMedicine.

[36] Hülse R., Biesdorf A., Hörmann K., Stuck B., Erhart M., Hülse M., Wenzel A. (2019). Peripheral Vestibular Disorders: An Epidemiologic Survey in 70 Million Individuals. Otol. Neurotol..

[37] Neuhauser H., Von Brevern M., Radtke A., Lezius F., Feldmann M., Ziese T., Lempert T. (2005). Epidemiology of vestibular vertigo A neurotologic survey of the general population. Neurology.

[38] Bisdorff A., Bosser G., Gueguen R., Perrin P. (2013). The epidemiology of vertigo, dizziness, and unsteadiness and its links to co-morbidities. Front. Neurol..

[39] Navari E., Cerchiai N., Casani A.P. (2018). Assessment of vestibulo-ocular reflex gain and catch-up saccades during vestibular rehabilitation. Otol. Neurotol..

[40] Chern A., Famuyide A.O., Moonis G., Lalwani A.K. (2021). Bilateral Sudden Sensorineural Hearing Loss and Intralabyrinthine Hemorrhage in a Patient With COVID-19. Otol. Neurotol..

[41] Koumpa F.S., Forde C.T., Manjaly J.G. (2020). Sudden irreversible hearing loss post COVID-19. BMJ Case Rep..

[42] Malayala S.V., Raza A. (2020). A case of COVID-19-induced vestibular neuritis. Cureus.

[43] Jha N.K., Ojha S., Jha S.K., Dureja H., Singh S.K., Shukla S.D., Chellappan D.K., Gupta G., Bhardwaj S., Kumar N. (2021). Evidence of Coronavirus (CoV) Pathogenesis and Emerging Pathogen SARS-CoV-2 in the Nervous System: A Review on Neurological Impairments and Manifestations. J. Mol. Neurosci..

[44] Furman J.M., Jacob R.G. (2001). A clinical taxonomy of dizziness and anxiety in the otoneurological setting. J. Anxiety Disord..

[45] Elibol E. (2021). Otolaryngological symptoms in COVID-19. Eur. Arch. Otorhinolaryngol..

[46] Viola P., Ralli M., Pisani D., Malanga D., Sculco D., Messina L., Laria C., Aragona T., Leopardi G., Ursini F. (2021). Tinnitus and equilibrium disorders in COVID-19 patients: Preliminary results. Eur. Arch. Otorhinolaryngol..

[47] Jafari Z., Kolb B.E., Mohajerani M.H. (2021). Hearing Loss, Tinnitus, and Dizziness in COVID-19: A Systematic Review and Meta-Analysis. Can. J. Neurol. Sci..

[48] Beukes E.W., Baguley D.M., Jacquemin L., Lourenco M., Allen P.M., Onozuka J., Stockdale D., Kaldo V., Andersson G., Manchaiah V. (2020). Changes in Tinnitus Experiences During the COVID-19 Pandemic. Front. Public Health.

[49] Freni F., Meduri A., Gazia F., Nicastro V., Galletti C., Aragona P., Galletti C., Galletti B., Galletti F. (2020). Symptomatology in head and neck district in coronavirus disease (COVID-19): A possible neuroinvasive action of SARS-CoV-2. Am. J. Otolaryngol..

[50] Alves de Sousa F., Pinto Costa R., Xara S., Nobrega Pinto A., Almeida E.S.C. (2021). SARS-CoV-2 and hearing: An audiometric analysis of COVID-19 hospitalized patients. J. Otol..

[51] Sia S.F., Yan L.M., Chin A.W.H., Fung K., Choy K.T., Wong A.Y.L., Kaewpreedee P., Perera R., Poon L.L.M., Nicholls J.M. (2020). Pathogenesis and transmission of SARS-CoV-2 in golden hamsters. Nature.

[52] Delmas B., Laude H. (1990). Assembly of coronavirus spike protein into trimers and its role in epitope expression. J. Virol.

[53] Matsuyama S., Nagata N., Shirato K., Kawase M., Takeda M., Taguchi F. (2010). Efficient activation of the severe acute respiratory syndrome coronavirus spike protein by the transmembrane protease TMPRSS2. J. Virol.

[54] Ziegler C.G.K., Allon S.J., Nyquist S.K., Mbano I.M., Miao V.N., Tzouanas C.N., Cao Y., Yousif A.S., Bals J., Hauser B.M. (2020). SARS-CoV-2 Receptor ACE2 Is an Interferon-Stimulated Gene in Human Airway Epithelial Cells and Is Detected in Specific Cell Subsets across Tissues. Cell.

[55] Wang H., Zhou M., Brand J., Huang L. (2009). Inflammation and taste disorders: Mechanisms in taste buds. Ann. N. Y. Acad. Sci..

[56] Meinhardt J., Radke J., Dittmayer C., Franz J., Thomas C., Mothes R., Laue M., Schneider J., Brunink S., Greuel S. (2020). Olfactory transmucosal SARS-CoV-2 invasion as a port of central nervous system entry in individuals with COVID-19. Nat. Neurosci..

[57] Benghanem S., Mazeraud A., Azabou E., Chhor V., Shinotsuka C.R., Claassen J., Rohaut B., Sharshar T. (2020). Brainstem dysfunction in critically ill patients. Crit. Care.

[58] Nouchi A., Chastang J., Miyara M., Lejeune J., Soares A., Ibanez G., Saadoun D., Morelot-Panzini C., Similowski T., Amoura Z. (2021). Prevalence of hyposmia and hypogeusia in 390 COVID-19 hospitalized patients and outpatients: A cross-sectional study. Eur. J. Clin. Microbiol. Infect. Dis..

[59] Brandal L.T., MacDonald E., Veneti L., Ravlo T., Lange H., Naseer U., Feruglio S., Bragstad K., Hungnes O., Odeskaug L.E. (2021). Outbreak caused by the SARS-CoV-2 Omicron variant in Norway, November to December 2021. Euro Surveill..

[60] Iacobucci G. (2021). COVID-19: Runny nose, headache, and fatigue are commonest symptoms of omicron, early data show. BMJ.

