# Supplementary material for: Post-COVID-19 Impairment of the Senses of Smell, Taste, Hearing, and Balance
Source: Viruses. 2022 Apr 20;14(5):849. doi: 10.3390/v14050849 (PMC9145380; doi:10.3390/v14050849)
Supplement: Supplementary file 1 [file viruses-14-00849-s001.zip › viruses-1642871-supplementary.pdf]

Supplementary Material:

Supplementary Table S1 Subjective and objective impairment by smell, taste, vertigo

	**During COVID-19** Mean (95% CI)		**After COVID-19** Mean (95% CI)		**P value**
**Olfactory impairment (VAS)**	7.5 (6.4-8.7)		3.0 (2.1-3.8)		*<0.0001*
**Taste impairment (VAS)**	6.9 (5.8-8.0)		1.7 (1.0-2.4)		*<0.0001*
**Vertigo (DHI)**	16.0 (10.5-21.5)		5.1 (2.5-7.6)		*<0.0001*
	**During COVID-19** Mean (95% CI)		**After COVID-19** Mean (95% CI)		**P value**
			**1-3M**	**4-6M**	
**Olfactory impairment - Sniffing sticks**	N/A		28.0 (26.6-29.4)	27.3 (23.3-31.2)	>0.05
**Taste impairment – Taste Test**	N/A		92.7 (85.2-100.3)	94.4 (89.1-99.8)	>0.05
**Vertigo (vHIT right)**	N/A		1.0 (0.97-1.1)	1.1 (0.98-1.15)	>0.05
**Vertigo (vHIT left)**	N/A		1.0 (0.98-1.1)	1.0 (0.95-1.1)	>0.05
	**During COVID-19** Mean (95% CI)		**After COVID-19** Mean (95% CI)		**P value**
	**Male**	**Female**	**Male**	**Female**	
**Olfactory impairment (VAS)**	6.5 (4.5-8.4)	8.3 (7.1-9.6)	2.5 (1.3-3.8)	3.3 (2.1-4.5)	>0.05
**Taste impairment (VAS)**	6.0 (4.1-7.8)	7.6 (6.2-9.0)	1.7 (0.6-2.7)	1.7 (0.7-2.7)	>0.05
**Vertigo (DHI)**	11.1 (3.0-19.3)	19.9 (12.4-27.5)	2.3 (0.3-4.4)	7.7 (3.6-11.8)	>0.05
**Olfactory impairment - Sniffing sticks (TDI)**	N/A		29.4 (27.5-31.4)	26.4 (23.9-28.7)	*<0.05*
**Taste impairment – Taste Test**	N/A		98.9 (96.7-101.2)	88.5 (79.3-97.6)	0.035
**Vertigo (vHIT right)**	N/A		1.1 (0.99-1.2)	1.0 (0.97-1.1)	>0.05

**Vertigo (vHIT left)**	N/A	1.1 (0.99-1.1)	1.0 (0.96-1.1)	>0.05

**Supplementary Table S2 Correlation analysis of questionnaires & clinical tests**		
	Spearman correlation	P value
**Olfactory impairment (VAS) &**		
**Threshold**	-0.3	0.07
**Discrimination**	-0.4	*0.003*
**Identification**	-0.4	*0.004*
**TDI score**	-0.5	*0.001*
**Taste impairment (VAS) & Taste test**	0.3	0.08
**Vertigo (DHI) &**		
**vHIT right**	-0.03	0.8
**vHIT left**	0.1	0.4

**Supplementary Table S3 Frequency-specific analysis of pure tone audiograms**	
**Bone conduction**	Audiometric test
**Low frequencies (0.25-0.5 kHz)**	
Normal hearing (0-20 dB)	40 (80)
Mild (20-40 dB)	2 (4)
Medium (40-60 dB)	0 (0)
Severe (>60 dB)	0 (0)
No data/Pre-existing	8 (16)
Total Mean ± SD	9.6 ± 4.97
**Medium frequencies (1-4 kHz)**	
Normal hearing (0-20 dB)	34 (68)
Mild (20-40 dB)	8 (16)
Medium (40-60 dB)	0 (0)
Severe (>60 dB)	0 (0)
No data/Pre-existing	8 (16)
Total Mean ± SD	11.92 ± 7.63
**High frequencies (6 kHz)**	
Normal hearing (0-20 dB)	34 (68)
Mild (20-40 dB)	5 (10)
Medium (40-60 dB)	0 (0)
Severe (>60 dB)	2 (4)
No data/Pre-existing	9 (18)
Total Mean ± SD	13.94 ± 16.75
**Air conduction**	

**Low frequencies (0.125-0.5 kHz)**	
Normal hearing (0-20dB)	34 (68)
Mild (20-40 dB)	8 (16)
Medium (40-60 dB)	0 (0)
Severe (>60 dB)	0 (0)
No data/Pre-existing	8 (16)
Total Mean ± SD	15.94 ± 5.88
**Medium frequencies (1-4 kHz)**	
Normal hearing (0-20dB)	29 (58)
Mild (20-40 dB)	13 (26)
Medium (40-60 dB)	0 (0)
Severe (>60 dB)	0 (0)
No data/Pre-existing	8 (16)
Total Mean ± SD	16.72 ± 8.13
**High frequencies (6-10 kHz)**	
Normal hearing (0-20dB)	26 (52)
Mild (20-40 dB)	10 (20)
Medium (40-60 dB)	4 (8)
Severe (>60 dB)	2 (4)
No data/Pre-existing	8 (16)
Total Mean ± SD	22.62 ± 16.34

Figure S1

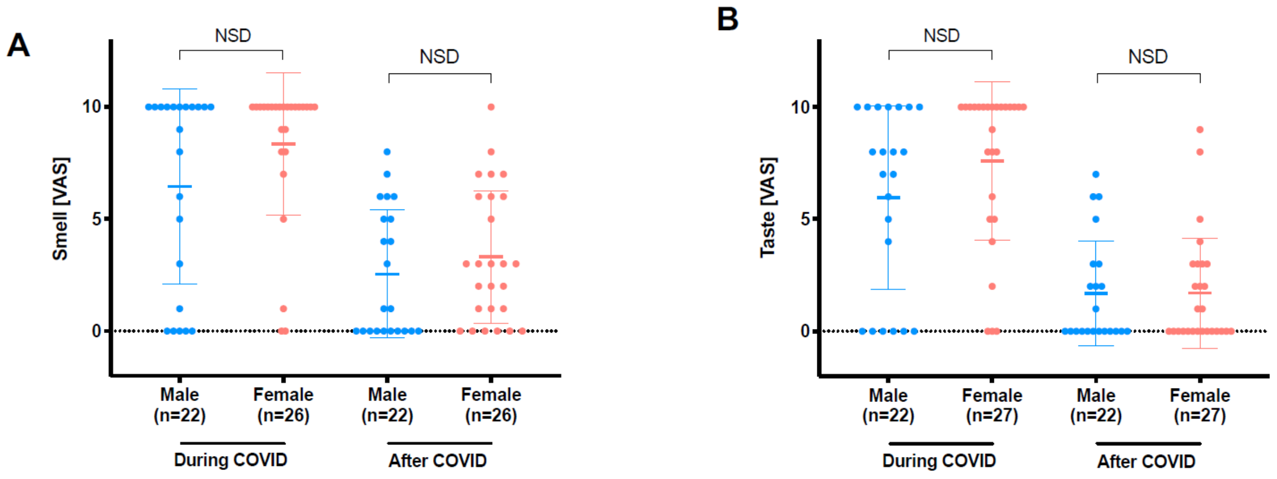

Supplementary Figure S1. The subjective impairment of smell and taste distinguished by gender. Subjective evaluations of smell (A) and taste (B) did not differ between men and women (NSD: no significant data).

Figure S2

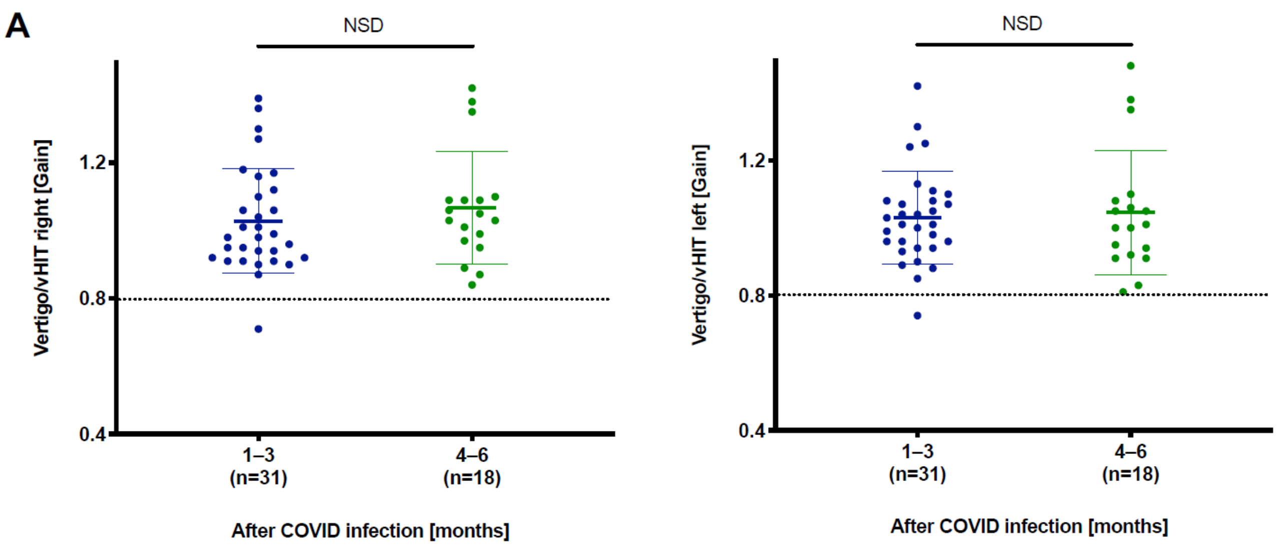

Supplementary Figure S2. Video head impulse test (vHIT) after COVID infection. In the follow-up period, all patients were tested for affection of the semicircular canal using vHIT. The right (A) and left (B) sides of patients were tested for gain. Gain reduction of the right or left vestibular system was detected in one patient in the initial three months following COVID-19.
